# The Link between Posttraumatic Stress Disorder and Functionality among United States Military Service Members Psychiatrically Hospitalized Following a Suicide Crisis

**DOI:** 10.3390/healthcare6030095

**Published:** 2018-08-07

**Authors:** Sissi Palma Ribeiro, Jessica M. LaCroix, Fernanda De Oliveira, Laura A. Novak, Su Yeon Lee-Tauler, Charles A. Darmour, Kanchana U. Perera, David B. Goldston, Jennifer Weaver, Alyssa Soumoff, Marjan Ghahramanlou-Holloway

**Affiliations:** 1 Suicide Care, Prevention, and Research Initiative, Department of Medical & Clinical Psychology, Uniformed Services University, Bethesda, MD 20814, USA; spribeiro3@gmail.com (S.P.R.); jessica.lacroix.ctr@usuhs.edu (J.M.L.); fernanda.de-oliveira@usuhs.edu (F.D.O.); laura.novak.ctr@usuhs.edu (L.A.N.); su-yeon.lee-tauler.ctr@usuhs.edu (S.Y.L.-T.); charles.darmour.ctr@usuhs.edu (C.A.D.); kanchana.perera.ctr@usuhs.edu (K.U.P.); 2 Department of Psychiatry, Duke University, Durham, NC 27708, USA; david.goldston@duke.edu; 3 Inpatient Psychiatry, Fort Belvoir Community Hospital, VA 22060, USA; jennifer.j.weaver6.civ@mail.mil; 4 Department of Psychiatry, Walter Reed National Military Medical Center, Bethesda, MD 20889, USA; alyssa.a.soumoff.mil@mail.mil

**Keywords:** PTSD, functionality, suicide, comorbidity, military, inpatient

## Abstract

Posttraumatic stress disorder (PTSD) is one of the most commonly diagnosed psychiatric disorders in the United States and has been linked to suicidal thoughts and behaviors, yet the role of a PTSD diagnosis on functional impairment among suicidal individuals remains unknown. This study examined the association between PTSD status and functional impairment among military psychiatric inpatients admitted for acute suicide risk (*N* = 166) with a lifetime history of at least one suicide attempt. Measures of functionality included: (1) alcohol use; (2) sleep quality; (3) social problem-solving; and (4) work and social adjustment. Thirty-eight percent of the sample met criteria for PTSD. Women were more likely than men to meet criteria for PTSD (*p* = 0.007), and participants who met PTSD criteria had significantly more psychiatric diagnoses (*p* < 0.001). Service members who met PTSD criteria reported more disturbed sleep (*p* = 0.003) and greater difficulties with work and social adjustment (*p* = 0.004) than those who did not meet PTSD criteria. However, functionality measures were not significantly associated with PTSD status after controlling for gender and psychiatric comorbidity. Gender and number of psychiatric comorbidities other than PTSD were significant predictors of PTSD in logistic regression models across four functionality measures. Future studies should assess the additive or mediating effect of psychiatric comorbidities in the association between impaired functioning and PTSD. Clinicians are encouraged to assess and address functionality during treatment with suicidal individuals, paying particular attention to individuals with multiple psychiatric diagnoses.

## 1. Introduction

Posttraumatic stress disorder (PTSD) and suicide are serious health concerns among U.S. military service members and veterans. In a recent meta-analysis by Fulton and colleagues [1], the overall prevalence of PTSD among Operation Enduring Freedom (OEF)/Operation Iraqi Freedom (OIF) veterans was estimated at 23%. Among over 100,000 returning Iraq and Afghanistan veterans, 25% received at least one mental health diagnosis, and more than half of those were diagnosed with PTSD (52%) [2]. The link between PTSD and suicidal thoughts and behaviors is well-documented among military samples [3,4,5,6,7], and suicide is currently a leading cause of death among active duty U.S. military personnel [8]. According to the most recent Department of Defense Suicide Event Report (DoDSER) [9], PTSD was among the most common psychiatric diagnoses for individuals with a history of suicide attempt; two-thirds of active duty personnel with a documented suicide attempt received at least one mental health diagnosis, 20% of whom received a PTSD diagnosis.

One potential mechanism by which PTSD and suicide may be linked is through impaired functioning. The diathesis-stress model has been applied to several psychiatric diagnoses when describing how biological, historical, and environmental factors impact functionality before and after adverse events. Specifically, the diathesis-stress model has been used to conceptualize the development of psychiatric disorders, such as PTSD and Major Depressive Disorder (MDD), as the result of events and circumstances that surpass the individual’s ability to cope [10]. Previous research among civilians diagnosed with PTSD indicate that functional impairment may manifest in the forms of disrupted sleep patterns [11], poor social support [12], and increased likelihood of divorce [13]. Functional impairment may in turn be associated with increased risk for suicide. For instance, among individuals who had planned or attempted suicide, 82% reported severe or extreme life impairment, compared to 70% among individuals who had suicide ideation, and 37% among individuals with no suicide ideation [14].

Similar links between PTSD and functioning related to social domains, alcohol use, and sleep difficulties have been found in military samples. One longitudinal study found a strong predictive relationship between PTSD and decreased social support over time, suggesting that interpersonal functioning difficulties associated with PTSD symptoms may erode the quality and quantity of social support resources [15]. Among individuals with PTSD, interpersonal difficulties may come about when social support networks are perceived as dangerous and unreliable [16]. As support networks are viewed with frustration, members are deemed unsafe, and social interactions may reintroduce elements of the traumatic exposure [17]. Over time, trauma-exposed individuals may avoid social relationships to increase perceived safety, thereby negatively affecting their social bonds. Additionally, in an analysis of data collected from veterans diagnosed with PTSD, researchers found evidence that those who reported no available social support networks were at greater risk of suicidal ideation and behaviors [18]. Thus, the relation between PTSD symptoms and interpersonal difficulties, including social problem-solving difficulties and social support, may indicate an individual’s limited functionality. 

Alcohol use among military members diagnosed with PTSD is common and potentially problematic, with excess alcohol consumption often leading to military service related occupational and legal issues [19]. In a study of 596 combat veterans, problematic alcohol use was estimated in approximately 39% of the sample [20]. A study of 205 OEF/OIF combat veterans indicated that alcohol-related consequences partially mediated the association between PTSD and quality of life [21]. Moderate to high alcohol use over 30 years of follow-up period was associated with adverse structural brain outcomes including hippocampal atrophy [22]. Cognitive impairment associated with alcohol misuse may impede recovery from alcohol or PTSD treatment, due to reduced ability to comprehend treatment materials or not being able to fully practice the strategies learned for treatment success [23].

In military service members, sleep disturbances are common and are often the result of rotating work schedules, work stress, and deployment related events [24]. Sleep problems are also a feature of PTSD [25] and may contribute to the maintenance of PTSD symptomatology [26]. Sleep deprivation is thought to affect basic arousal systems such as the limbic system [27] and prevent extinction of conditioned fear learning [28]. These disturbances may indicate decreased functionality and performance in a host of cognitive and emotional areas [29,30], which may prevent treatment, hinder work and life balance, and put the service member at risk for other taxing outcomes such as additional psychiatric diagnoses.

Overall, several researchers have stressed the importance of identifying correlates related to functionality [31,32]. Despite the recent influx of literature on PTSD, we continue to lack understanding on the association between functional impairment and PTSD among individuals at elevated risk of suicide. One important question that remains unexplored is whether functional impairment among military service members with acute suicide risk have greater likelihood of having a PTSD diagnosis, regardless of gender and number of psychiatric comorbidities.

The current study sought to examine the association between PTSD status and functional impairment among military psychiatric inpatients admitted for a recent suicide crisis and at least one lifetime suicide attempt. Functionality was explored using a functional measure for work and social adjustment, as well as proxy measures investigating alcohol use, sleep quality, and social problem-solving abilities. We hypothesized that individuals who met criteria for PTSD would report greater alcohol use, more sleep problems, greater social problem-solving difficulties, and more work and social impairments, compared to individuals who did not meet PTSD criteria.

## 2. Materials and Methods

### 2.1. Participants and Procedures

Participants were military personnel, at least 18 years of age, who were psychiatrically hospitalized at an inpatient military treatment facility following a suicide crisis (i.e., a recent suicide attempt or suicide ideation requiring admission to an inpatient unit; *N* = 166). Participants were recruited within 72 h of admission. All participants had a history of at least one lifetime suicide attempt defined as “a potentially self-injurious behavior with a nonfatal outcome for which there is evidence, either explicit or implicit, that the individual intended to kill himself or herself” [33]. Data were collected as part of an ongoing randomized controlled trial evaluating the efficacy of Post-Admission Cognitive Therapy (PACT) to reduce suicide risk among psychiatric inpatients [34,35]. Participants were voluntarily admitted to the inpatient unit, able to provide informed consent, cognitively capable of completing psychological assessments, able to communicate in English, and were cleared by the inpatient treatment team to participate. Cross-sectional baseline data were used for this study.

### 2.2. Measures

All measures used in this study were administered as part of the baseline assessment for the larger randomized controlled trial. Self-report measures were administered by a bachelor’s or master’s level research case manager. Clinician-administered measures were administered by a doctoral-level research clinician. The current study used a functional measure for work and social adjustment, the Work and Social Adjustment Scale (WSAS), as well as proxy measures of functionality to assess alcohol use, sleep quality, and social problem-solving abilities. These measures were added as proxy measures of functionality given the association of increased alcohol use [36,37], poor sleep quality [38], and social problem-solving difficulties [39] with PTSD symptomatology and functioning deficits.

#### 2.2.1. Alcohol Use Disorders Identification Test (AUDIT)

The AUDIT is a 10-item, clinician-administered measure that screens for excessive drinking. Items are rated on a 5-point Likert scale, with higher total scores indicating greater alcohol use and greater alcohol-related impairment. The AUDIT has been tested in a variety of settings and populations and has demonstrated high internal consistency and test-retest reliability. The internal consistency of the AUDIT for the current sample showed a Cronbach’s α of 0.89 [40].

#### 2.2.2. Columbia Suicide Severity Rating Scale (C-SSRS)

The C-SSRS is a clinician-administered measure of lifetime intensity of suicide ideation and behavior. Assessed behaviors include preparatory behavior and interrupted, aborted, and actual suicide attempts based on definitions by the Centers for Disease Control and Prevention [41].

#### 2.2.3. Mini International Neuropsychiatric Screen and Interview (MINI)

The MINI is a brief, structured, clinician-administered assessment of psychiatric disorders corresponding to the Diagnostic and Statistical Manual, Fourth Edition, Text Revision (DSM-IV-TR), including PTSD (Module I) [42]. The PTSD module is composed of six dichotomous questions (I1 through I6) which explore current and past experiences of traumatic events according to the DSM-IV-TR criteria. PTSD criteria were assessed based on questions pertaining to lifetime and primarily current (i.e., past month) experience of PTSD symptoms. A “Yes/No” coding was assigned to each participant based on whether they met criteria for a current PTSD diagnosis. Note that the MINI does not provide information regarding PTSD symptom severity [43].

#### 2.2.4. Pittsburgh Sleep Quality Index (PSQI)

The PSQI is a 19-item self-report measure that assesses overall sleep quality and patterns within the past month. Higher scores indicate more impaired sleep. The PSQI has demonstrated high internal consistency (Cronbach’s α = 0.83), global score test-retest reliability, and good sensitivity and specificity in distinguishing between good and poor sleepers [44].

#### 2.2.5. Social Problem-Solving Inventory-Revised, Short Form (SPSI-R:S)

The SPSI-R:S is a 25-item self-report measure that assesses participants’ problem-solving abilities. Questions are rated on a 5-point Likert scale, with responses ranging from 0 (“not at all true of me”) to 4 (“extremely true of me”). Lower scores on the SPSI-R:S indicate worse problem-solving abilities. For the current study, the SPSI-R:S demonstrated a Cronbach’s α of 0.67 [45].

#### 2.2.6. Work and Social Adjustment Scale (WSAS)

The WSAS is a 5-item self-report questionnaire that measures the impact of an individual’s mental health on their ability to work and perform daily tasks effectively. Items are rated on a 9-point Likert scale ranging from 0 (“not at all impaired”) to 8 (“severely impaired”). Higher scores on the WSAS indicate greater impairment in work and social adjustment. The WSAS demonstrated good internal consistency with the current sample (Cronbach’s α = 0.86) [46].

### 2.3. Statistical Analysis

Initial analyses were conducted using between-subjects *t*-tests and chi-square tests to compare participants who did and did not meet PTSD criteria on demographic variables and functional outcomes of interest. Additionally, Cohen’s *d* was computed to provide another form of presenting the association between functionality measures and PTSD. Logistic regression analyses were then conducted to further explore the association between functional measures and PTSD status adjusting for data-driven covariates (i.e., gender and psychiatric comorbidities).

## 3. Results

### 3.1. Demographic Characteristics

Detailed demographic information is presented in Table 1. Participants were predominately male (65%), and Caucasian (65%), and nearly half were married (43%). The average age was 29.8 years old (*SD* = 8.8). All participants had a history of at least one lifetime suicide attempt, and 62% reported a lifetime history of multiple suicide attempts. Overall, 38% (*n* = 63) of participants met criteria for PTSD. Women were more likely than men to meet criteria for PTSD (52% vs. 48%; *p* = 0.007), and participants who met PTSD criteria had significantly more psychiatric diagnoses than those who did not meet PTSD criteria (*M* = 6.37, *SD* = 2.30 vs. *M* = 3.66, *SD* = 2.30).

### 3.2. Functional Differences

A series of *t*-tests using Bonferroni adjusted α levels of 0.0125 per test (i.e., 0.05/4) were conducted to investigate between-group differences on each of the functionality measures. Participants who met criteria for PTSD had significantly higher level of impaired sleep as measured by PSQI (*p* = 0.003), and significantly greater impairment in work and social adjustment as measured by the WSAS (*p* = 0.004), compared to participants who did not meet the criteria for PTSD. No statistically significant differences between groups were found on the AUDIT or the SPSI-R:S (Table 2).

### 3.3. Logistic Regression Results

Logistic regression analyses were conducted to assess for the effects of functional measures on the odds of participants meeting the PTSD criteria after controlling for gender and number of additional psychiatric disorders. Results indicated that higher scores on any functional measures were not associated with meeting the PTSD criteria after controlling for gender and number of additional psychiatric disorders (Table 3). Female gender significantly predicted meeting criteria for PTSD in all four models. Similarly, a greater number of additional psychiatric diagnoses was predictive of meeting PTSD criteria in all models.

## 4. Discussion

The current study examined the extent to which functionality was associated with meeting diagnostic criteria for PTSD (versus not meeting PTSD criteria) in a sample of psychiatric inpatients with an acute suicide crisis and at least one lifetime attempted suicide. We found that more than one third (38%) of the sample of patients psychiatrically hospitalized for acute suicide ideation or attempt met criteria for PTSD. Women and patients with higher psychiatric comorbidities were more likely to meet PTSD criteria. While individuals with PTSD had greater impairment in sleep and work and social adjustment, the associations between functional measures and PTSD were no longer significant when adjusting for gender and number of psychiatric diagnoses. Number of psychiatric comorbidities and gender were significant predictors of meeting PTSD criteria for all four of our functionality measures.

### 4.1. Demographics

While it is estimated that between 14% and 16% percent of deployed veterans and military personnel have been diagnosed with PTSD [47], little is known about the prevalence of PTSD in military inpatient settings. The current study found that 97% of participants met criteria for at least one psychiatric diagnosis (separate analysis) and over a third of them met criteria for PTSD. This result corroborates previous data reporting the occurrence of PTSD and psychiatric comorbidities [2,48,49,50]. Notably, this finding supports current concerns about a lack of trauma-focused services offered to high-risk individuals, such as those with a history of a suicide attempt and psychiatric comorbidities [51]. Historically, most clinical trials testing the efficacy of evidence-based treatments for PTSD, such as Cognitive Processing Therapy (CPT) and Prolonged Exposure (PE) therapy, have been known to exclude participants with suicidal thoughts and behaviors, resulting in a dearth of knowledge on how to best treat individuals at high-risk of suicide with PTSD [52].

Additionally, the current study found that women were more likely than men to meet PTSD criteria, corroborating the findings of previous studies [53]. A study by Kang and colleagues [54] reported that, while women in the military generally have lower rates of combat exposure, they report higher rates of military sexual trauma, which has been shown to have a strong association with the development of PTSD. Similarly, a meta-analysis of 290 studies found that women are more likely than men to meet criteria for PTSD despite being less likely to experience potentially traumatic events, with the exception of sexual assault or abuse [53]. While the current study did not take a particular focus on gender differences or the types of trauma reported by participants, it is important to consider that the results could be associated with extraneous factors, such as that women are more likely to seek help for mental health-related disorders [55] and are more willing to disclose distressing information to others [56]. On the other hand, mental health stigma is generally higher among men, especially for those who have been sexually assaulted, thus increasing the chances of underreporting trauma [57].

### 4.2. PTSD, Functionality and Comorbidity

Initial analyses demonstrated that, compared to participants who did not meet criteria for PTSD, participants who met criteria for PTSD were significantly more likely to report sleep difficulties and greater impairment in work and social adjustment. The significant association of PTSD criteria and functional deficits in sleep and work and social adjustment has been robustly supported in previous studies [15,58]. We did not find significant differences on alcohol use or social problem-solving abilities across PTSD status. Because our sample was characterized by both high suicide risk and psychiatric comorbidities, functionality measures such as alcohol use and social problem-solving skills may not have been noticeably different between those with and without PTSD.

After controlling for gender and psychiatric comorbidity, however, there was no significant association between the measures of functional impairment and the outcome of PTSD status. Instead, female gender and increased psychiatric comorbidities were associated with higher likelihood of meeting criteria for PTSD. Further analyses are necessary to determine if psychiatric comorbidities are stronger predictors of PTSD status or if psychiatric comorbidities mediate or moderate the association between functional measures and PTSD.

Psychiatric comorbidity is common among people who die by suicide [58,59]. While depressive and substance use disorders have the strongest association with increased suicide risk, PTSD, insomnia, poor sleep quality, and nightmares have also been found to increase risk of suicide in multiple studies [60,61]. Veterans who screened positive for PTSD and two or more psychiatric diagnoses were significantly more likely to endorse suicidal ideation than veterans with PTSD alone [5]. Further, a case-control analysis of 5.4 million citizens in Denmark found that those diagnosed with PTSD and depression showed additive risk for suicide compared to those diagnosed with either disorder alone [61], and studies on alcohol use disorder have found similar results [62]. This study and our findings highlight the importance of psychiatric comorbidity in relation to PTSD status, above and beyond functionality among individuals with heightened suicide risk.

### 4.3. Limitations and Strengths

Several limitations should be considered when interpreting results. First, this study presents baseline data analysis of a larger study assessing the efficacy of the PACT treatment for suicide thoughts and behaviors that was not originally intended to assess functionality in relation to PTSD status. The WSAS was the only measure in this study specifically designed to assess functionality, and is a short, 5-item self-report measure that is not considered a gold standard assessment of functionality. The AUDIT, PSQI, and SPSI-R:S are measures for investigating important clinical domains that have a functional impact on the overall well-being of a patient but were not intentionally designed to assess functional domains. Previous studies have used the Outcome Questionnaire-45 (OQ-45) [63] and the 36-Item Short Form Health Survey [64] when investigating functional outcomes. Both measures are valid, reliable, and easy to administer in a clinical or research setting. Functionality has been mostly studied for other psychiatric diagnoses, such as MDD and bipolar disorder [65,66,67].

Furthermore, the strengths of associations in our current study may have be limited due to restricting our study to a high-risk, highly comorbid inpatient sample with elevated functional impairments. As previously mentioned, all participants in the current study were presented to the inpatient psychiatric unit following a suicide-related crisis and had a lifetime history of at least one suicide attempt. Results and interpretations should not be extended to those without a lifetime history of suicide attempt. Similarly, the results of the current study should not be extended to all members of the United States military as only a subset of the military population has acute suicide risk. In addition, our use of cross-sectional data from an RCT study made it impossible to assess causality between PTSD status and functional impairment.

The current study benefited from the inclusion of a widely used, reliable, clinician-administered diagnostic measure, the MINI. Many studies rely on retrospective chart reviews or self-report assessments to determine psychiatric diagnoses, which, particularly in settings where the individual’s full psychiatric history is not accessible (e.g., emergency departments, inpatient facilities, and other settings outside of an individual’s usual mental health care network), may lead to inaccurate data. The MINI is one of the most widely used assessment tools for the screening of psychiatric diagnoses and has been found to have good validity and reliability [42]. This assessment allowed for more standardized data gathering. However, it is important to note that the MINI was developed to be administered as a screener, acting as a decision support tool, and is meant to be used in conjunction with a clinical interview and other diagnostic tools.

Despite these limitations, findings from the current study may inform assessment and treatment of individuals psychiatrically hospitalized following a suicide-related crisis and have implications for future research on the link between functionality and PTSD among individuals with acute suicidal thoughts and behaviors.

### 4.4. Implications

It is important for clinicians to assess functionality throughout treatment. For over thirty years, the American Psychiatric Association has included a section in the Diagnostic and Statistical Manual (DSM) entirely focused on the assessment of one’s functionality, called the Global Assessment of Functioning Scale (GAF; Axis V) [43]. In 2013, the new DSM dropped the multiaxial organization, along with the separate section for the assessment of functioning, opting instead for the recommendation that providers consider a new tool, the World Health Organization Disability Assessment Schedule 2.0 (WHODAS 2.0). As Gold described in her recent review [68], this new measure makes a conceptual distinction between medical and mental health conditions, and the disabilities resulting from them. This was found to be a notable improvement with important clinical implications, bringing more focus to mental health conditions and better assessment of important functional variables when assessing a patient’s quality of life. Clinicians and patients can benefit from the implementation of functional assessments in clinical and research settings. The current study adds to a growing body of literature examining functional domains in relation to PTSD and suicide thoughts and behaviors.

### 4.5. Recommendations for Future Research

In addition to addressing the limitations described above, future studies should consider assessing additive or mediating effects of psychiatric comorbidity on PTSD and suicidal thoughts and behaviors. As mentioned previously, veterans with PTSD who screened positive for two other diagnoses were 5.7 times more likely to endorse suicide ideation than those with a PTSD diagnosis alone [5]. This comorbidity, in turn, may place the individual at a higher risk of suicide. For individuals with comorbid psychiatric conditions, it is important to investigate the role of each psychiatric diagnosis and the combinations of psychiatric comorbidities in increasing functional impairments and suicide risk. 

Furthermore, future studies should assess whether specific types of functional impairments (in addition to the ones investigated in this study) are associated with PTSD and increased risk for suicide. For example, PTSD has been shown to have an indirect effect on suicidal ideation through increased interpersonal conflict, apprehension, and decreased perceived family support [69]. Functional impairments should also be considered during the development and administration of psychotherapy by monitoring comprehensive constructs of functionality and addressing functionality as a mechanism to improve patients’ well-being.

## 5. Conclusions

The current study provides further evidence on the notable rates of PTSD and psychiatric comorbidity in a sample of psychiatric inpatients with at least one lifetime suicide attempt. Psychiatrically hospitalized individuals with a history of suicide attempt who met criteria for PTSD reported more disturbed sleep and greater difficulty in the ability to work and perform daily activities effectively. However, the association between functionality and PTSD status was no longer significant after adjusting for gender and psychiatric comorbidity. Notably, the vast majority of the sample met criteria for at least one psychiatric diagnosis and those with a greater number of psychiatric comorbidities demonstrated higher likelihood of meeting PTSD criteria. Future studies should consider investigating the causal pathways in which multiple medical and psychiatric comorbidities are associated with functional deterioration and PTSD outcomes. 

## Figures and Tables

**Table 1:** Demographic and military service characteristics of military psychiatric inpatients hospitalized following a suicide crisis (*N* = 166).

Characteristic	*N*	%
**Age (*M, SD*), Years**	29.8	8.8
**Gender**		
Male	108	65.1
Female	58	34.9
**Race**		
American Indian or Alaska Native	3	1.8
Asian	10	6.0
Black or African American	31	18.7
Native Hawaiian or Other Pacific Islander	2	1.2
White or Caucasian	107	64.5
Two or More Races	12	7.2
Other	1	0.6
**Marital Status**		
Single	62	37.3
Married	72	43.4
Separated/Divorced	22	19.2
**Service Branch**		
Air Force	17	10.2
Army	75	45.2
Coast Guard	1	0.6
Marine Corps	30	18.1
Navy	43	25.9
**Rank**		
E-1–E-4	74	44.6
E-5–E-8	66	39.8
O-1–O-5	17	10.2
W-2–W-3	3	1.8
Cadet/Midshipmen	6	3.6
**Deployment History**		
No	79	47.6
Yes	85	51.2
**Combat Deployment**		
No	26	15.7
Yes	58	34.9
Not Applicable	79	47.6

**Table 2:** Mean difference in functional impairment measures across participants with lifetime suicide attempt histories who did or did not meet PTSD criteria.

Measures	*N*	*M (SD)*		Cohen’s *d*	*t*	*p*
		**Met PTSD Criteria** ***n* = 63**	**Did Not Meet PTSD Criteria** ***n* = 103**			
Alcohol Use Disorders Identification Test (AUDIT)	166	8.33 (9.00)	6.82 (7.86)	−0.18	1.14	0.255
Pittsburgh Sleep Quality Index (PSQI)	165	13.74 (3.42)	11.80 (4.30)	−0.49	3.03	0.003
Social Problem-Solving Inventory-Revised, Short Form (SPSI-R:S) ^1^	166	95.49 (16.59)	94.51 (17.70)	−0.06	0.35	0.724
Work and Social Adjustment Scale (WSAS)	166	28.29 (8.16)	23.76 (10.47)	−0.47	2.93	0.004

^1^ SPSI-R:S: age adjusted scores.

**Table 3:** The odds of meeting PTSD criteria by functional measures (i.e., alcohol, sleep, social problem-solving, and work and social adjustment) adjusting for gender and number of other psychiatric diagnoses.

Measures	OR	95% CI	*p*
**Alcohol Use Disorders Identification Test (AUDIT)**			
AUDIT Total Score	0.992	1	0.748
Male (Reference = female)	0.378	1	0.018
n other psychiatric diagnoses	1.636	1	<0.001
**Pittsburgh Sleep Quality Index (PSQI)**			
PSQI Total Score	1.045	1	0.378
Male (Reference = female)	0.352	1	0.008
n other psychiatric diagnoses	1.578	1	<0.001
**Social Problem-Solving Inventory-Revised, Short Form (SPSI-R:S) ^1^**			
SPSI-R:S Total Score	1.023	1	0.050
Male (Reference = female)	0.363	1	0.010
n other psychiatric diagnoses	1.690	1	<0.001
**Work and Social Adjustment Scale (WSAS)**			
WSAS Total Score	1.011	1	0.622
Male (Reference = female)	0.374	1	0.013
n other psychiatric diagnoses	1.604	1	<0.001

^1^ SPSI-R:S: age adjusted scores.

## References

[1] Fulton J.J., Calhoun P.S., Wagner H.R., Schry A.R., Hair L.P., Feeling N., Beckham J.C. (2015). The prevalence of posttraumatic stress disorder in Operation Enduring Freedom/Operation Iraqi Freedom (OEF/OIF) veterans: A meta-analysis. J. Anxiety Disord..

[2] Seal K.H., Bertenthal D., Miner C.R., Sen S., Marmar C.R. (2007). Mental health disorders among 103,788 US veterans returning from Iraq and Afghanistan seen at Department of Veterans Affairs facilities. Arch. Intern. Med..

[3] Bryan C.J., Corso K.A. (2011). Depression, PTSD, and suicidal ideation among active duty veterans in an integrated primary care clinic. Psychol. Serv..

[4] Guerra V.S., Calhoun P.S. (2011). Examining the relation between posttraumatic stress disorder and suicidal ideation in an OEF/OIF veteran sample. J. Anxiety Disord..

[5] Jakupcak M., Cook J., Imel Z., Rosenheck R., McFall M. (2009). PTSD as a Risk Factor for Suicidal Ideation in Iraq and Afghanistan War Veterans. J. Trauma Stress.

[6] Pietrzak R.H., Goldstein M.B., Malley J.C., Rivers A.J., Johnson D.C., Southwick S.M. (2010). Risk and protective factors associated with suicidal ideation in veterans of Operations Enduring Freedom and Iraqi Freedom. J. Affect. Disord..

[7] Ramsawh H.J., Fullerton C.S., Mash H.H., Ng T.H., Kessler R.C., Stein M.B., Ursano R.J. (2014). Risk for suicidal behaviors associated with PTSD, depression, and their comorbidity in the U.S. Army. J. Affect. Disord..

[8] Armed Forces Health Surveillance Center (AFHSC) (2014). Surveillance snapshot: Manner and cause of death, active component, US Armed Forces, 1998–2013. MSMR.

[9] Pruitt L.D., Smolenski D.J., Bush N.E., Skopp N.A., Hoyt T.V., Grady B.J. Department of Defense Suicide Event Report (DoDSER) Calendar Year 2015 Annual Report. http://t2health.dcoe.mil/programs/dodser.

[10] McKeever V.M., Huff M.E. (2003). A diathesis-stress model of posttraumatic stress disorder: Ecological, biological, and residual stress pathways. Rev. Gen. Psychol..

[11] Dell’Osso L., Massimetti G., Conversano C., Bertelloni C.A., Carta M.G., Ricca V., Carmassi C. (2014). Alterations in circadian/seasonal rhythms and vegetative functions are related to suicidality in DSM-5 PTSD. BMC Psychiatry.

[12] Kotler M., Iancu I., Efroni R., Amir M. (2001). Anger, impulsivity, social support, and suicide risk in patients with posttraumatic stress disorder. J. Nerv. Ment. Dis..

[13] Maia D.B., Marmar C.R., Metzler T., Nóbrega A., Berger W., Mendlowicz M.V., Coutinho E.S., Figueira I. (2007). Post-traumatic stress symptoms in an elite unit of Brazilian police officers: Prevalence and impact on psychosocial functioning and on physical and mental health. J. Affect. Disord..

[14] Tarrier N., Gregg L. (2004). Suicide risk in civilian PTSD patients: Predictors of suicidal ideation, planning and attempts. Soc. Psychiatry. Epidemiol..

[15] King D.W., Taft C., King L.A., Hammond C., Stone E.R. (2006). Directionality of the association between social support and posttraumatic stress disorder: A longitudinal investigation. J. Appl. Soc. Psychol..

[16] Ehlers A., Clark D.M. (2000). A cognitive model of posttraumatic stress disorder. Behav. Res. Ther..

[17] Price M., Gros D.F., Strachan M., Ruggiero K.J., Acierno R. (2013). The role of social support in exposure therapy for Operation Iraqi Freedom/Operation Enduring Freedom veterans: A preliminary investigation. Psychol. Trauma.

[18] Jakupcak M., Vannoy S., Imel Z., Cook J.W., Fontana A., Rosenheck R., McFall M. (2010). Does PTSD moderate the relationship between social support and suicide risk in Iraq and Afghanistan War Veterans seeking mental health treatment?. Depress. Anxiety.

[19] Stahre M.A., Brewer R.D., Fonseca V.P., Naimi T.S. (2009). Binge drinking among US active-duty military personnel. Am. J. Prev. Med..

[20] Eisen S.V., Schultz M.R., Vogt D., Glickman M.E., Elwy A.R., Drainoni M., Martin J. (2012). Mental and physical health status and alcohol and drug use following return from deployment to Iraq or Afghanistan. Am. J. Public Health.

[21] Angkaw A.C., Haller M., Pittman J.E., Nunnink S.E., Norman S.B., Lemmer J.A., Baker D.G. (2015). Alcohol-related consequences mediating PTSD symptoms and mental health-related quality of life in OEF/OIF combat veterans. Mil. Med..

[22] Topiwala A., Allan C.L., Valkanova V., Zsoldos E., Filippini N., Sexton C., Mahmood A., Fooks P., Singh-Manoux A., Mackay C.E. (2017). Moderate alcohol consumption as risk factor for adverse brain outcomes and cognitive decline: Longitudinal cohort study. BMJ.

[23] Allen D.N., Goldstein G., Seaton B.E. (1997). Cognitive rehabilitation of chronic alcohol abusers. Neuropsychol. Rev..

[24] Peterson A.L., Goodie J.L., Satterfield W.A., Brim W.L. (2008). Sleep disturbance during military deployment. Mil. Med..

[25] American Psychiatric Association (2013). Diagnostic and Statistical Manual of Mental Disorders: DSM-5.

[26] Gilbert K.S., Kark S.M., Gehrman P., Bogdanova Y. (2015). Sleep disturbances, TBI and PTSD: Implications for treatment and recovery. Clin. Psychol. Rev..

[27] Rauch S.L., Whalen P.J., Shin L.M., McInerney S.C., Macklin M.L., Lasko N.B., Pitman R.K. (2000). Exaggerated amygdala response to masked facial stimuli in posttraumatic stress disorder: A functional MRI study. Biol. Psychiatry.

[28] Wessa M., Flor H. (2007). Failure of extinction of fear responses in posttraumatic stress disorder: Evidence from second-order conditioning. Am. J. Psychiatry.

[29] Killgore W.D., Balkin T.J., Wesensten N.J. (2006). Impaired decision making following 49 h of sleep deprivation. J. Sleep Res..

[30] Pilcher J.J., Huffcutt A.J. (1996). Effects of sleep deprivation on performance: A meta-analysis. Sleep.

[31] Pietrzak R.H., Johnson D.C., Goldstein M.B., Malley J.C., Rivers A.J., Morgan C.A., Southwick S.M. (2010). Psychosocial buffers of traumatic stress, depressive symptoms, and psychosocial difficulties in veterans of Operations Enduring Freedom and Iraqi Freedom: The role of resilience, unit support, and postdeployment social support. J. Affect. Disord..

[32] Thomas J.L., Wilk J.E., Riviere L.A., McGurk D., Castro C.A., Hoge C.W. (2010). Prevalence of mental health problems and functional impairment among Active Component and National Guard soldiers 3 and 12 months following combat in Iraq. Arch. Gen. Psychiatry.

[33] O’Carroll P.W., Berman A.L., Maris R.W., Moscicki E.K., Tanney B.L., Silverman M.M. (1996). Beyond the tower of Babel: A nomenclature for suicidology. Suicide Life Threat Behav..

[34] Ghahramanlou-Holloway M., Cox D., Greene F. (2012). Post-admission cognitive therapy: A brief intervention for psychiatric inpatients admitted after a suicide attempt. Cogn. Behav. Pract..

[35] Ghahramanlou-Holloway M., Neely L.L., Tucker J. (2014). A cognitive behavioral strategy for preventing suicide. Curr. Psychiatry.

[36] Grossbard J.R., Hawkins E.J., Lapham G.T., Williams E.C., Rubinsky A.D., Simpson T.L., Seal K.H., Kivlahan D.R., Bradley K.A. (2013). Follow-up care for alcohol misuse among OEF/OIF veterans with and without alcohol use disorders and posttraumatic stress disorder. J. Subst. Abuse Treat..

[37] Sannibale C., Teesson M., Creamer M., Sitharthan T., Bryant R.A., Sutherland K., Taylor K., Bostock-Matusko D., Visser A., Peek-O’Leary M. (2013). Randomized controlled trial of cognitive behaviour therapy for comorbid post-traumatic stress disorder and alcohol use disorders. Addiction.

[38] Mohr D., Vedantham K., Neylan T., Metzler T.J., Best S., Marmar C.R. (2003). The mediating effects of sleep in the relationship between traumatic stress and health symptoms in urban police officers. Psychosom. Med..

[39] Hofmann S.G., Litz B.T., Weathers F.W. (2003). Social anxiety, depression, and PTSD in Vietnam veterans. J. Anxiety Disord..

[40] Babor T.F., Higgins-Biddle J.C., Saunders J.B., Monteiro M.G., World Health Organization (WHO) The Alcohol Use Disorders Identification Test: Guidelines for Use in Primary Care. http://apps.who.int/iris/bitstream/handle/10665/67205/WHO_MSD_MSB_01.6a.pdf;jsessionid=46F1535BD74886CCD130D5E5FDE9B3BF?sequence=1.

[41] Posner K., Brown G.K., Stanley B., Brent D.A., Yershova K.V., Oquendo M.A., Currier G.W., Melvin G.A., Greenhill L., Shen S. (2011). The Columbia–Suicide Severity Rating Scale: Initial validity and internal consistency findings from three multisite studies with adolescents and adults. Am. J. Psychiatry.

[42] American Psychiatric Association (2000). DSM-IV-TR: Diagnostic and Statistical Manual of Mental Disorders, Text Revision.

[43] Sheehan D.V., Lecrubier Y., Sheehan K.H., Janavs J., Weiller E., Keskiner A., Schinka J., Knapp E., Sheehan M.F., Dunbar G.C. (1997). The validity of the Mini International Neuropsychiatric Interview (MINI) according to the SCID-P and its reliability. Eur. Psychiatry.

[44] Buysse D.J., Reynolds C.F., Monk T.H., Berman S.R., Kupfer D.J. (1989). The Pittsburgh Sleep Quality Index: A new instrument for psychiatric practice and research. Psychiatry Res..

[45] D’Zurilla T.J., Nezu A.M., Maydeu-Olivares A. (2002). Social Problem-Solving Inventory—Revised (SPSI-R).

[46] Mundt J.C., Marks I.M., Shear M.K., Greist J.M. (2002). The Work and Social Adjustment Scale: A simple measure of impairment in functioning. Br. J. Psychiatry.

[47] Gates M.A., Holowka D.W., Vasterling J.J., Keane T.M., Marx B.P., Rosen R.C. (2012). Posttraumatic stress disorder in veterans and military personnel: Epidemiology, screening, and case recognition. Psychol. Serv..

[48] Stein M.B., McQuaid J.R., Pedrelli P., Lenox R., McCahill M.E. (2000). Posttraumatic stress disorder in the primary care medical setting. Gen. Hosp. Psychiatry.

[49] Brown P.J., Stout R.L., Mueller T. (1999). Substance use disorder and posttraumatic stress disorder comorbidity: Addiction and psychiatric treatment rates. Psychol. Addict. Behav..

[50] Kessler R.C., Sonnega A., Bromet E., Hughes M., Nelson C.B. (1995). Posttraumatic stress disorder in the National Comorbidity Survey. Arch. Gen. Psychiatry.

[51] Bryan C.J., Clemans T.A., Hernandez A.M., Mintz J., Peterson A.L., Yarvis J.S., Resick P.A. (2016). Evaluating potential iatrogenic suicide risk in trauma-focused group cognitive behavioral therapy for the treatment of PTSD in active duty military personnel. Depress. Anxiety.

[52] Bakalar J.L., Carlin E.A., Blevins C.L., Ghahramanlou-Holloway M. (2016). Generalizability of evidence-based PTSD psychotherapies to suicidal individuals: A review of the Veterans Administration and Department of Defense clinical practice guidelines. Mil. Psychol.

[53] Tolin D.F., Foa E.B. (2006). Sex differences in trauma and posttraumatic stress disorder: A quantitative review of 25 years of research. Psychol. Bull..

[54] Kang H., Dalager N., Mahan C., Ishii E. (2005). The role of sexual assault on the risk of PTSD among Gulf War veterans. Ann. Epidemiol..

[55] Wang P.S., Aguilar-Gaxiola S., Alonso J., Angermeyer M.C., Borges G., Bromet E.J., Bruffaerts R., De Girolamo G., De Graaf R., Gureje O. (2007). Use of mental health services for anxiety, mood, and substance disorders in 17 countries in the WHO world mental health surveys. Lancet.

[56] Ward M., Tedstone Doherty D., Moran R. (2007). It’s Good to Talk: Distress Disclosure and Psychological Wellbeing.

[57] Vogel D.L., Wade N.G. (2009). Stigma and help-seeking. Psychologist.

[58] Babson K.A., Feldner M.T. (2010). Temporal relations between sleep problems and both traumatic event exposure and PTSD: A critical review of the empirical literature. J. Anxiety Disord..

[59] Conner K.R., Bohnert A.S., McCarthy J.F., Valenstein M., Bossarte R., Ignacio R., Lu N., Ilgen M.A. (2013). Mental disorder comorbidity and suicide among 2.96 million men receiving care in the Veterans Health Administration health system. J. Abnorm. Psychol..

[60] Ribeiro J.D., Pease J.L., Gutierrez P.M., Silva C., Bernert R.A., Rudd M.D., Joiner T.E. (2012). Sleep problems outperform depression and hopelessness as cross-sectional and longitudinal predictors of suicidal ideation and behavior in young adults in the military. J. Affect. Disord..

[61] Gradus J.L., Qin P., Lincoln A.K., Miller M., Lawler E., Sørensen H.T., Lash T.L. (2010). Posttraumatic stress disorder and completed suicide. Am. J. Epidemiol..

[62] Flensborg-Madsen T., Knop J., Mortensen E.L., Becker U., Sher L., Grønbæk M. (2009). Alcohol use disorders increase the risk of completed suicide—Irrespective of other psychiatric disorders. A longitudinal cohort study. Psychiatry Res..

[63] Lambert M.J., Burlingame G.M., Umphress V., Hansen N.B., Vermeersch D.A., Clouse G.C., Yanchar S.C. (1996). The reliability and validity of the Outcome Questionnaire. Clin. Psychol. Psychother..

[64] Ware J.E., Sherbourne C.D. (1992). The MOS 36-item short-form health survey (SF-36): I. Conceptual framework and item selection. Med. Care.

[65] Reznik A.E., Sudharshan L., Stephens J.M., Shelbaya A., Pappadopulos E., Haider S., Lin I., Gao C. (2015). Impact of Major depressive Disorder on Patient functionality and work performance in Emerging Markets. Value Health.

[66] Prelipceanu D., Purnichi T., Marinescu V., Matei V. (2015). The daily functionality in a major depressive episode cohort of Romanian patients—A non-interventional study. Maedica.

[67] Esposito-Smythers C., Goldstein T., Birmaher B., Goldstein B., Hunt J., Ryan N., Keller M. (2010). Clinical and psychosocial correlates of non-suicidal self-injury within a sample of children and adolescents with bipolar disorder. J. Affect. Disord..

[68] Gold L.H. (2014). DSM-5 and the assessment of functioning: The World Health Organization Disability Assessment Schedule 2.0 (WHODAS 2.0). J. Am. Acad. Psychiatry Law Online.

[69] Dutton C.E., Rojas S.M., Badour C.L., Wanklyn S.G., Feldner M.T. (2016). Posttraumatic stress disorder and suicidal behavior: Indirect effects of impaired social functioning. Arch. Suicide Res..

